# Development of a Suicide Prediction Model for the Elderly Using Health Screening Data

**DOI:** 10.3390/ijerph181910150

**Published:** 2021-09-27

**Authors:** Seo-Eun Cho, Zong Woo Geem, Kyoung-Sae Na

**Affiliations:** 1 Department of Psychiatry, Gachon University College of Medicine, Gil Medical Center, Incheon 21565, Korea; arztin01@gilhospital.com; 2 College of IT Convergence, Gachon University, Seongnam 13120, Korea; zwgeem@gmail.com

**Keywords:** suicide, the elderly, health screening cohort, machine learning, mental health

## Abstract

Suicide poses a serious problem globally, especially among the elderly population. To tackle the issue, this study aimed to develop a model for predicting suicide by using machine learning based on the elderly population. To obtain a large sample, the study used the big data health screening cohort provided by the National Health Insurance Sharing Service. By applying a machine learning technique, a predictive model that comprehensively utilized various factors was developed to select the elderly aged > 65 years at risk of suicide. A total of 48,047 subjects were included in the analysis. Individuals who died by suicide were older, and the number of men was significantly greater. The suicide group had a more prominent history of depression, with the use of medicaments significantly higher. Specifically, the prescription of benzodiazepines alone was associated with a high suicide risk. Furthermore, body mass index, waist circumference, total cholesterol, and low-density lipoprotein level were lower in the suicide group. We developed a model for predicting suicide by using machine learning based on the elderly population. This suicide prediction model can satisfy the performance to some extent by employing only the medical service usage behavior without subjective reports.

## 1. Introduction

Suicide is a serious social and cultural problem in modern society. According to the World Health Organization (WHO), over 700,000 people die by suicide every year [1]. Thus, the goal of the WHO Mental Health Action Plan 2013–2030 was to reduce the suicide rate in all WHO member states by one-third before 2030 [2].

Suicide among the elderly has emerged as a serious health and social problem in both the East and the West. The age group with the highest suicide rate in the United States in 2019 included individuals aged > 85 years, of which 21.12 of every 100,000 people died by suicide [3]. Furthermore, this group is the only age group for which the suicide rate has increased, considering the 2018 rate (19.07). The suicide rate for the elderly is also high in South Korea. Moreover, Korea has one of the highest suicide rates among the members of the Organization for Economic Co-operation and Development. According to the most recent data, the average suicide rate in OECD countries is 11.2, and South Korea ranked first with 23.0, followed by former Soviet states such as Lithuania (22.2) and Latvia (18.1). The country with the second highest suicide rate in Asia is Japan at 14.9 [4]. In 2019, the suicide rate was 46.6 per 100,000 people aged ≥ 65 years in South Korea, which is more than twice the average suicide rate and the highest among all age groups [5].

Older people are prone to loneliness and economic poverty. Unlike young adults and middle-aged people, they encounter difficulties forming active interpersonal relationships because people close to them continue to die or are already dead, and they experience repeated loss. Moreover, they become more vulnerable to economic factors as economic activities become difficult. In addition, the quality of life is reduced due to various chronic diseases, such as high blood pressure, diabetes, arthritis, dysuria, and cancer, and the elderly feel greater physical discomfort. Usually, when they feel that the pain they are experiencing is intolerable, unchanging, and endless, they consider suicide as a way out [6]. From this perspective, factors such as interpersonal relationships, economic difficulties, physical illness, and functional decline all contribute to an increasing suicide rate. In a previous study on the elderly in Korea, the important factors leading to suicide attempts in suicidal ideation were male sex, education level, and age discrimination [7].

As the problem of suicide among the elderly is widespread and serious, prevention and preemptive interventions are required. In previous studies [8,9], suicide prevention interventions in the elderly population were found to reduce suicidal thoughts and suicide rates. For this reason, interest in the elderly suicide model has increased, including the development of protocols for models that describe and predict suicide in the elderly [10]. Thus, one of the common methods is to use a measurement tool. However, no measure has yet been proven to be effective in predicting future suicide. In fact, the United Kingdom’s National Institute for Health and Care Excellence guideline recommends not to use risk assessment instruments to predict future suicide, suicide attempts, or self-harm [11]. For the elderly, expressing feelings in a way that is easy for others to understand can be also difficult, like in cases of masked depression [12]. In addition, several suicide measures include many questions. There are many elders who cannot use such measures as smoothly as younger adults, due to deterioration of vision and cognitive ability because of aging. Thus, models predicting the suicide risk among the elderly should fully consider this.

Due to the fact that suicide is a very rare event, an approach taking this into account is required. In some cases, groups for which suicide is more common than for the general population, such as individuals suffering from depression or bipolar disorder, are selected for studies [13]. In other cases, a suicide attempt instead of suicide is used as a key outcome indicator [14]. However, both approaches have limitations. Limiting subjects to a clinical population only prevents the inclusion of subjects who do not fit into the therapeutic system. Furthermore, although a suicide attempt is the single largest risk factor for suicide, and is most similar to suicide, suicide attempts and suicide have distinct characteristics [15,16].

Accordingly, the best method is to predict suicide in the general population of a community. A previous study on the suicide prediction model [17], which included 2,960,929 subjects from 2009 to 2015, was limited in that only outpatients from the department of psychiatric medicine were included. In a systematic review [18] of studies that included 64 unique suicide prediction models, the general population was not included. The predictive validity of suicide was very low in most studies and practical utility was limited. As is widely known, a study of community-dwelling suicidal ideators over 65 years of age found that both depression and stress predict the intensity of suicidal thoughts [19]. There has also been a study [20] showing that both big data and machine learning methods need to be improved for accurate suicide prediction.

The biggest challenge in predicting suicide in the general population is the need to obtain a large sample. For this, the authors used a big data health screening dataset provided by the National Health Insurance Sharing Service (NHISS) [21]. In addition, in a socio-cultural environment with a high social prejudice against suicide, such as in Korea, there is a limit to suicide prevention because predictions and interventions are limited to groups at high risk of suicide. Therefore, using health check-up data that are generally available for the entire population, it is possible to reduce problems such as avoidance due to stigma. By applying a machine-learning technique, a predictive model that comprehensively utilizes various factors was developed to select the elderly with a high risk of suicide.

## 2. Materials and Methods

### 2.1. Data and Subjects

We used the health screening cohort database (DB) provided by the NHISS. Ten percent of the 5.15 million people eligible for health insurance aged between 40 and 79 years as of the end of December 2002, among those who undertook general health screening between 2002 and 2003, were selected by a simple random sampling technique. Qualifications and income information (socioeconomic variables), hospital and clinic usage history, health screening results, and healthcare institution information from 2002 to 2015 (14 years) for 514,866 people were constructed in a cohort format. These data were constructed anonymously in a cohort format so that these data can be used for research purposes in South Korea [21].

We then specifically targeted patients aged ≥ 65 years who undertook health screening in 2009 from the health screening cohort DB, and this was done for several reasons. Firstly, various measurement variables and questionnaire items have changed since 2008 and 2009, resulting in inconsistency. Secondly, since the subjects were older than 65, more people die over time, and thus, the subjects at the baseline of the analysis inevitably become scarcer. Accordingly, those who took health screening in 2009, which was the earliest possible period, were targeted.

This study was approved by the institutional review board (IRB No. GDIRB2019-253) of the Gil Medical Center.

### 2.2. Variables

We used the following variables in the study: age, sex, disability, medical benefits, diagnosis of depressive disorder in the past year as of the date of examination, psychiatric drug use in the past year (antidepressants, mood stabilizers, sleeping pills, benzodiazepines, anti-psychotic drugs, and cognitive enhancement drugs), ranking of insurance premium payment at the time of examination, body mass index (BMI), waist circumference, systolic blood pressure, fasting plasma glucose, total cholesterol, triglyceride, creatinine, hemoglobin, aspartate transaminase, and gamma-glutamyl transpeptidase. All psychiatric diagnoses were in accordance with the *International Statistical Classification of Diseases and Related Health Problems 10th Revision* (ICD-10) [22]. When the diagnosis fell under F32 and F33, it was defined as a depressive disorder. Antidepressants included citalopram, escitalopram, paroxetine, fluoxetine, fluvoxamine, venlafaxine, desvenlafaxine, duloxetine, milnacipran, bupropion, mirtazapine, tianeptine, trazodone, clomipramine, amitriptyline, imipramine, amoxapine, and moclobemide. Mood stabilizers included lithium, lamotrigine, valproic acid (or divalproex), and carbamazepine. Antipsychotic drugs included chlorprothixene, haloperidol, levomepromazine, molindone, perphenazine, pimozide, sulpiride, amisulpride, aripiprazole, blonanserin, clozapine, olanzapine, quetiapine, risperidone, ziprasidone, and zotepine. Sleeping pills included zolpidem and doxepine. Benzodiazepines included flunitrazepam, flurazepam, chlordiazepoxide, clobazam, triazolam, clonazepam, diazepam, lorazepam, mexazolam, bromazepam, clotiazepam, alprazolam, and etizolam. Cognitive enhancers included donepezil, galantamine, rivastigmine, and memantine.

According to the common recommendations of academia [23] and various studies [17,24,25], cases falling under the ICD-10 codes X60–X84 or Y10–Y34 were defined as suicide. The tracking period for suicide was until 2015.

### 2.3. Data Preprocessing and Machine Learning

The extraction of data necessary for research from the entire health screening cohort DB was carried out using the SAS Enterprise Guide 7.1 (SAS Institute Inc., Cary, NC, USA). All cases with missing values were excluded from the analysis. All subsequent work was carried out by using R 3.3.0 [26] and RStudio 1.0.136 [27]. In addition, all data were divided into a training set (0.7) and a test set (0.3) at a ratio of 7:3, respectively. Considering that suicide is very rare, the synthetic minority over-sampling technique was used [28]. No structural transformation of the data took place in the test set. The entire training dataset was trained within the training set through 10-fold cross-validation. The area under the receiver operating characteristic curve (AUC) was established as the main performance indicator. In addition, an overall accuracy, sensitivity, specificity, negative predictive value, and positive predictive value were calculated. Performance measurements, such as hyper-parameter tuning, confusion matrix composition, and AUC, were all performed using the *caret* library.

For the algorithm, random forest was used. In general, the decision tree model has the advantage that variable importance and classification mechanisms can be intuitively known and the computing cost is low. However, the decision tree is rarely used as an algorithm for solving problems in practice since it has the crucial problem of being vulnerable to overfitting. Random forest is the ensemble model to compensate for the shortcomings of this decision tree [29]. It maximizes predictive power when new data (i.e., test set) are given rather than used for training by minimizing the overfitting of decision tree. Thanks to this advantage, random forest has been used as a practical problem-solving algorithm in various fields [30,31].

**Figure 1:**
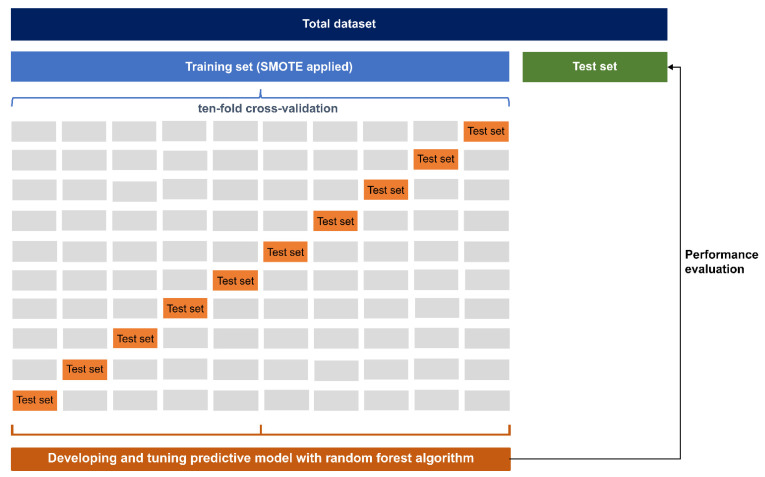
The overview of machine learning.

To find out which variables contributed significantly to optimization of the predictive model, we calculated variable importance by the principle of “mean decreases in accuracy” [32]. The process of estimating the importance of variable j is as follows: Several cases were not sampled and were called out-of-bag because bagging permits cases to be sampled more than once for the same classifier. The out-of-bag samples hand down to the tree, and the prediction accuracy then decreases when training by utilizing one of the trees is performed. Subsequently, the variable j was changed in the out-of-bag samples at random, and the accuracy was calculated again. Before and after the permutation over all trees, the raw score for the importance of variable j was calculated by averaging the gap in out-of-bag errors. Subsequently, the score was returned to normal by utilizing the standardized deviation of the difference. Eventually, the score was reduced so that the minimum value was set to 0 and the maximum value was set to 100. Using care, a confusion matrix, hyperparameter tuning, and performance measurement were attained. In brief, the prediction performance of the out-of-bag part of the data was recorded for each tree. Then, the prediction performance was measured and recorded each time, while the predictors were permuted alternatively. Based on all trees, the average value of the difference between these two prediction performances was obtained and then normalized according to the standard error. The final prediction performance was measured by using test data, which were never used in the training phase. An overall overview of machine learning is presented in Figure 1.

## 3. Results

### 3.1. Socio-Demographic and Clinical Characteristics

A total of 48,047 subjects were included in the analysis. Among them, the number of people who died by suicide was 100 (0.21%). Their sociodemographic and clinical characteristics are presented in Table 1. Those who died by suicide were older than those who did not, and significantly more men than women died by suicide. Among those who underwent health screening, the percentage of men and women was almost equal, but in the suicide group, 72% were men. Moreover, the suicide group had a greater history of depression and significantly higher use of antidepressants, mood stabilizers, antipsychotic drugs, sleeping pills, and benzodiazepines than the non-suicide group. Regarding examination results, BMI, waist circumference, total cholesterol, and low-density lipoprotein (LDL) levels were lower in the suicide group.

### 3.2. Prediction Model Performance and Variable Importance

**Figure 2:**
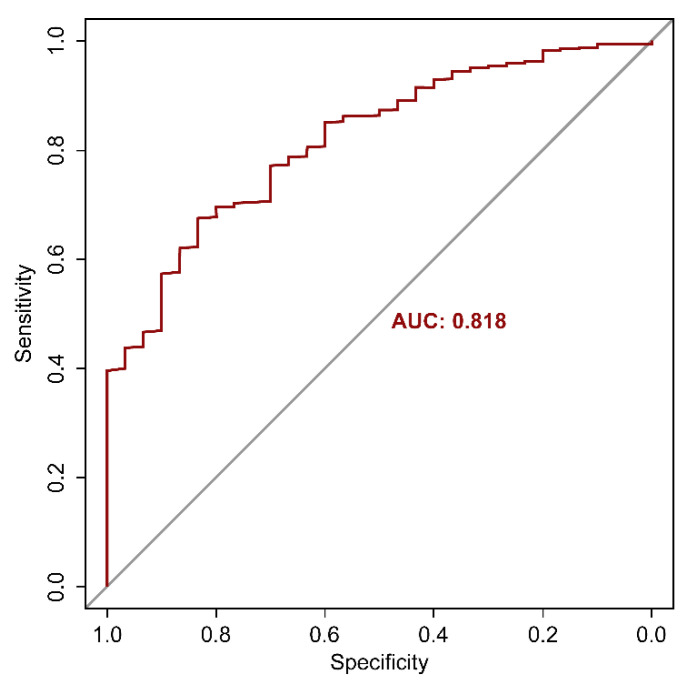
Area under the receiver operating characteristic curve of the suicide prediction model.

**Figure 3:**
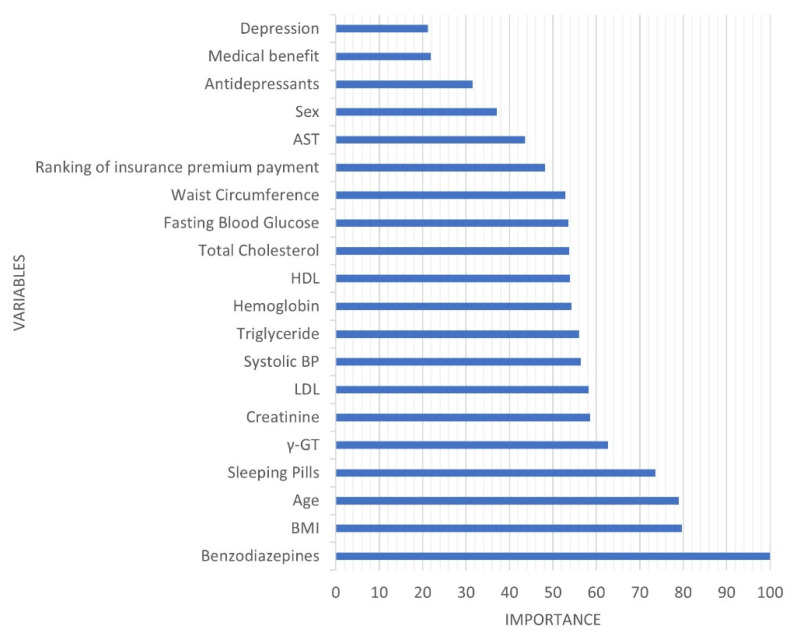
Variables’ importance in the suicide prediction model.

The AUC, which is the most preferred indicator of the prediction model, was 0.818 (Figure 2). In addition, overall accuracy was 0.832, sensitivity was 0.600, specificity was 0.833, negative predictive value was 0.999, and positive predictive value was 0.007. The variable with the highest importance was the history of taking benzodiazepines, followed by BMI, age, and history of taking sleeping pills (Figure 3).

## 4. Discussion

We developed a model to predict the high-risk group for suicide using machine learning algorithms for the elderly aged > 65 years who underwent health screening. The fact that benzodiazepines had the most importance in this study has great implications. Benzodiazepines had the highest statistic among all variables with the χ^2^ value of 44.761 in a simple frequency analysis between the suicide and non-suicide groups. This is consistent with the results of previous studies, according to which prescription of benzodiazepines alone is associated with a high risk of suicide, even if not used as a means of suicide [33,34]. The mechanism by which the prescribed benzodiazepines and sleeping pills, collectively known as sedatives and hypnotics, are associated with suicide in elderly remains unclear. First, a possibility of misusing the prescribed sedatives and hypnotics for suicidal purposes can be considered. However, substance misuse is not a factor highly associated with suicide deaths, although it is mainly used for suicide attempts [35]. In 2019, the means that had the greatest impact on the suicide rate of the elderly older than 65 in South Korea was hanging (51.0%), followed by falling (18.0%), and taking pesticides (16.1%). Drugs accounted for only 2.3% of all means of suicide [5]. Another possibility is that the elderly who were prescribed sedatives and hypnotics had greater anxiety, agitation, and tension. Unfortunately, it is difficult to directly determine whether the deceased elderly used prescribed sedatives and hypnotics as a means, and to what extent psychiatric symptoms were actually experienced, due to the design of this study.

Another characteristic of suicide victims is that they had lower weight and slimmer waistlines, and lower total cholesterol and LDL levels. Low BMI, waist circumference, and LDL are all associated with low total cholesterol levels. The results of studies which support the connection between low cholesterol and suicide have been reported for a long time [36,37]. Recent studies elucidated the genetic overlap between cholesterol metabolism and suicide [38]. Additionally, among the medical parameters, GGT, creatinine, and systolic blood pressure were of high importance. Although the t-test indicated no significant difference, the mean GGT and creatinine levels were higher in the suicide deaths than in the control group, and the systolic blood pressure was slightly lower. Higher GGT levels were associated with a higher risk of suicide as alcohol intake increased. Creatinine is more likely to appear higher as the kidney function is reduced, drugs are taken, or with older age. Furthermore, the higher the drug intake, the more severe the underlying disease may be. Stress and suicide rates have been consistently found to be highly correlated [39]. As previously reported [40], stress may cause strain on the kidneys. It has been found that low systolic blood pressure is associated with suicidal ideation in the general Korean population [41], which is consistent with the results of this study. If the systolic blood pressure is low, there may be a possibility of the perception that overall negative emotions and pain cannot be suppressed [42].

Regarding the performance of the prediction model, it had the performance of AUC 0.818 and accuracy 0.832. The AUC value criterion for excellence has not been clearly established. A satisfactory AUC value criterion may vary due to various factors, such as data distribution, research area, and design. Generally, a value > 0.7 is considered acceptable, a value ≥ 0.8 is good, and a value > 0.9 is excellent [43]. This study was designed as a retrospective cohort follow-up approach. Another study compared the effect sizes from several studies and conducted follow-up analysis. It stated that an AUC score of 0.714 corresponds to Cohen’s d of 0.8 [44]. A Cohen’s d > 0.8 is considered to indicate a large effect size [45]. However, the low positive predictive value is considered a task to be overcome in the future. The probability of suicide in the general population is very low, at about 0.00025. For this reason, a low positive predictive value has always posed a problem in studies predicting suicide [46]. A review reported that positive predictive values of measures evaluating suicide risk in people receiving primary healthcare ranged from 0.01 to 0.2 [47]. In another meta-analysis, the positive predictive values of the tool, which predicts suicide risk, were analyzed for psychiatric patients only [48]. As a result, it was found to be 0.055 (95% confidence interval [CI] 0.035–0.085) in the high-risk and 0.09 (95% CI 0.005–0.017) in the low-risk group. Although the positive predictive value obtained in this study is insufficient as a par value, it is 28 times higher than the suicide rate of the general population.

There are several limitations to this study. Firstly, we encountered a difficulty in elucidating the mechanism of the prediction model due to the design of the retrospective cohort study. According to ICD-10, detailed methods of suicide are to be classified as sensitive information and are not provided to researchers. Thus, the role of the prescribed drug was not fully examined. Secondly, clinical information highly related to suicide, such as the severity of psychiatric symptoms, was unavailable. Thirdly, the study was not free from the selection bias, which fundamentally excluded people who had not undergone health screening. For small-subject studies, prospective randomized controlled studies and study designs that include more clinical information highly relevant to suicide may be possible. However, there is also a limitation in that it is difficult to design a study as a result of suicide death, and since the number of participants is small, there may be selective bias and low reliability.

Based on the results obtained while compensating for these limitations, practical data can be provided for developing suicide prevention programs and seeking counseling approaches to effectively prevent suicide from the stage of suicidal thoughts. The suicide prediction model built using big data can be widely used in most areas, such as national health checkups, emergency-room-based follow-up management projects for suicide attempters, and outpatient and inpatient treatment at medical institutions. The effectiveness of suicide prevention projects can be increased by enabling additional interest and intervention in elderly suicide attempters, especially those with higher actual suicide risk, using machine learning algorithms. Additional studies for tailored approaches to individual subjects should be conducted in the future.

## 5. Conclusions

This study developed a model for predicting suicide that occurs infrequently by using machine learning based on the elderly population aged > 65 years from the community. This suicide prediction model can satisfy the performance to some extent by employing only the medical service usage behavior without subjective reports from the elderly, although the model has many aspects that need to be improved in terms of a positive predictive value. By continuing research in this area and addressing the limitations of this study, it will be possible to implement the suicide prediction model practically in real life. Additionally, in suicide research using big data machine learning methods, structural suicide risk assessment tools should be used, and both the assessment of suicide risk and analysis to predict the most appropriate treatment options for individual patients should be performed.

## Figures and Tables

**Table 1:** Comparison of sociodemographic and economic characteristics and medical use between suicide and non-suicide groups.

Variables	Non-Suicide (*n* = 47,947)	Suicide (*n* = 100)	t or χ^2^	*p*-Value
Age, years	72.1 ± 4.41	74.1 ± 5.27	−3.72	0.0003 ^a^
Sex, male	24,228 (50.53%)	72 (72%)	18.4	<0.0001 ^b^
Disability, existence	1024 (2.14%)	7 (7%)	11.245	0.0008 ^b^
Medical benefit, existence	189 (0.39%)	0 (0%)	0.393	0.529 ^b^
Depression diagnosis, existence	2445 (5.1%)	17 (17%)	28.071	<0.0001 ^b^
**Taking psychiatric drugs, existence**				
Antidepressants	4095 (8.54%)	23 (23%)	26.625	<0.0001 ^b^
Mood stabilizer	576 (1.2%)	4 (4%)	0.011	0.011 ^b^
Antipsychotics	472 (0.98%)	4 (4%)	0.002	0.002 ^b^
Sleeping pills	1806 (3.77%)	16 (16%)	<0.0001	<0.0001 ^b^
Benzodiazepines	13,755 (28.69%)	59 (59%)	44.761	<0.0001 ^b^
Cognitive enhancer	412 (0.86%)	0 (0%)	0.8667	0.352 ^b^
Body measurement				
BMI, kg/m2	23.8 ± 3.1	21.8 ± 2.94	6.8	<0.0001 ^a^
Waist circumference, cm	83.17 ± 8.33	80.97 ± 8.21	2.67	0.009 ^a^
Systolic blood pressure, mmHg	129.8 ± 16.01	128.3 ± 16.82	0.95	0.345 ^a^
Diastolic blood pressure, mmHg	78.04 ± 10.02	77.89 ± 10.57	0.15	0.879 ^a^
**Blood biochemical results**				
Fasting plasma glucose, mg/dL	102.6 ± 26.08	102.5 ± 2.11	0.05	0.958 ^a^
Total cholesterol, mg/dL	196.4 ± 38.76	185.7 ± 40.36	2.65	0.009 ^a^
Hemoglobin, g/dL	13.41 ± 1.45	13.27 ± 1.8	0.81	0.4225 ^a^
AST, U/L	26.55 ± 15.7	32.17 ± 36.99	−1.52	0.132 ^a^
ALT, U/L	22.63 ± 16.84	24.96 ± 24.61	−0.94	0.351 ^a^
GGT, U/L	34.09 ± 49.93	50.36 ± 89.42	−1.82	0.072 ^a^
TG, mg/dL	138.6 ± 82.15	131.2 ± 83.99	0.9	0.369 ^a^
HDL, mg/dL	55.17 ± 35.98	59.47 ± 55.59	−0.77	0.441 ^a^
LDL, mg/dL	116.1 ± 38.74	107.6 ± 39.08	2020	0.028 ^a^
Creatinine, mg/dL	1.05 ± 1.11	1.21 ± 1.13	−1.42	0.203 ^a^

Data are presented as the mean ± standard deviation or number (%). Abbreviations: SD, standard deviation; AST, aspartate transaminase; ALT, alanine transaminase; GGT, gamma-glutamyl transpeptidase; TG, triglyceride; HDL, high-density lipoprotein; LDL, low-density lipoprotein. Statistical tests were performed using Student’s *t*-test ^a^ and the chi-square test ^b^.

## Data Availability

The datasets used in this study are publicly available at the “National Health Insurance Sharing Service” at https://nhiss.nhis.or.kr/bd/ab/bdaba000eng.do (accessed on 15 July 2021).
